# Cost-Effectiveness of Hypochlorous Acid Preserved Wound Cleanser versus Saline Irrigation in Conjunction with Ultrasonic Debridement for Complex Wounds

**DOI:** 10.36469/001c.28429

**Published:** 2021-11-01

**Authors:** Peter J. Mallow, John M. Hiebert, Martin C. Robson

**Affiliations:** 1 Health Services Administration Xavier University; 2 Truman Medical Center University of Missouri-Kansas City; 3 Division of Plastic Surgery University of South Florida

**Keywords:** hypochlorous acid preserved wound cleanser, cost-effectiveness, wound, wound healing

## Abstract

**Objective:** Low-frequency ultrasound debridement with irrigation is an effective method of wound bed preparation. A recent clinical study compared hypochlorous acid preserved wound cleanser (HAPWOC) to saline and found HAPWOC to be a more effective adjunct to low frequency ultrasound debridement. However, HAPWOC has an added cost. The primary objective of this study was to assess the cost-effectiveness of HAPWOC as an irrigation modality with low-frequency ultrasound debridement for the treatment of severely complex wounds that were destined to be closed primarily via a flap. The secondary objective of this study was to estimate the number needed to treat (NNT) to avoid a wound-related complication and its expected cost per NNT.

**Methods:** A patient-level Monte-Carlo simulation model was used to conduct a cost-effectiveness analysis from the US health system perspective. All clinical data were obtained from a prospective clinical trial. Cost data were obtained from the publicly available data sources in 2021 US dollars. The effect measure was the avoidance of wound-related complications at 14-days post-debridement. The primary outcome was the incremental cost-effectiveness ratio (ICER), a measure of the additional cost per benefit. The secondary outcomes were the NNT and expected cost per NNT to avoid one complication (complementary to the ICER in assessing cost-effectiveness). Deterministic and probabilistic sensitivity analyses (PSA) were performed to gauge the robustness and reliability of the results.

**Results:** The ICER for HAPWOC versus saline irrigation was US90.85perwoundcomplicationavoided.TheexpectedincrementalcostperpatientinthestudyandeffectwasUS49.97 with 55% relative reduction in wound-related complications at day 14 post debridement procedure. The NNT and cost per NNT were 2 and US$99.94, respectively. Sensitivity analyses demonstrated that these results were robust to variation in model parameters.

**Conclusion:** HAPWOC was a cost-effective strategy for the treatment of complex wounds during ultrasonic debridement. For every two patients treated with HAPWOC, one complication was avoided.

## INTRODUCTION

Wound bed preparation (WBP) has been a holistic and structured approach to determine which wounds are healable and best practices to consider during treatment.^1^ For healable complex wounds, WBP promotes debridement as a means to promote healing and reduce the time to reepithelization. A key element of WBP is physical removal of necrotic tissue. In addition to the removal of necrotic tissue, a key benefit of debridement is the removal of bacterial laden tissue.^2,3^ For those complex wounds that can be definitively closed with primary closure techniques involving grafts, approximations, or pedicle flaps, the quality of the WBP is of paramount importance. Failures of such primary closures of severely complex wounds have substantial negative consequences for the patient and the health-care system.^3^ It is estimated that there are more than 8.2 million Medicare beneficiaries with these types of wounds per year, which cost over US$28 billion.^4^

One approach to debridement is the use of low-frequency ultrasound. This approach combines mechanical and sharp debridement with irrigation and has been shown to disperse biofilms and decrease time to healing.^5,6^ Saline is typically used for irrigation with low-frequency ultrasound; however, saline does not possess anti-bacterial preservative properties.^7^ Hypochlorous acid preserved wound cleanser (HAPWOC), Vashe Wound Solution, Urgo Medical North America, Fort Worth, Texas, has been shown to mechanically reduce bacteria levels and to be non-cytotoxic and non-irritating to the wound.^8^ A recent prospective clinical study found low-frequency ultrasound debridement with HAPWOC to be more effective than saline for complex wounds.^7^ The primary clinical outcome was the number of wounds that were successfully closed 14 days post ultrasound debridement.^7^ However, the economic value of low-frequency ultrasound debridement with HAPWOC for complex wounds given its added cost is unknown. Thus, the objective of this study was to determine the cost-effectiveness of HAPWOC compared to saline for use in ultrasonic debridement of complex wounds. The specific context was closure success or failure following definitive closure via surgical techniques following WBP. The secondary objective of this study was to estimate the number needed to treat (NNT) and the expected cost of the NNT.

## METHODS

### Study Population

The study population consisted of 17 adult patients with complex stage 3 or 4 wounds of multiple etiologies. A full description of the study population and treatment procedures was published by Hiebert and Robson (2016).^7^ The patients were randomly assigned to receive HAPWOC (9 patients) or saline irrigation (8 patients) during their low-frequency ultrasonic mechanical debridement procedure. All patients were evaluated on days 1, 7, and 14 post-procedure for wound healing and the presence of wound-related complications.

### Modeling Strategy & Verification

**Figure 1.**
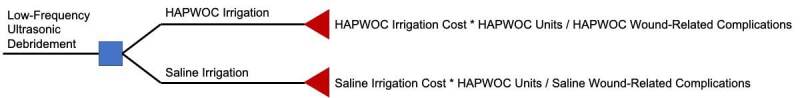
Model Diagram Abbreviations: HAPWOC, hypochlorous acid preserved wound cleanser. The model diagram illustrates the decision of using HAPWOC or Saline as the irrigation modality with low-frequency ultrasonic debridement of a complex wound.

A patient-level Monte-Carlo simulation model was developed to assess the cost-effectiveness of using HAPWOC versus saline irrigation for the treatment of complex wounds (Figure 1). The perspective of the model was the US health-care system. The model effectiveness measure was the avoidance of a wound-related complication at 14 days post-debridement procedure. The cost measures included the additional cost of HAPWOC for the debridement procedure. All other care was the same; thus, the costs were the same. All costs were reported in 2021 US dollars.

The primary outcome was the incremental cost effectiveness ratio (ICER). The ICER was calculated with the following formula:

$$\text{ICER}=\frac{\sum {\text{C}}_{\text{saline}}-\sum {\text{C}}_{\text{HAPWOC}}}{\sum {\text{Comp.}}_{\text{saline}}-\sum {\text{Comp.}}_{\text{HAPWOC}}}$$

Where: C_HAPWOC_ & C_saline_ were the summation of the total costs for HAPWOC and saline, respectively; Comp._HAPWOC_ & Comp._saline_ were the summation of the postoperative complications of HAPWOC and saline, respectively.

The NNT indicates the number of patients that need to be treated with HAPWOC to avoid one wound-related complication. It is a complementary measure to the ICER, which estimates how many individuals need to be treated to avoid one wound-related complication. Lower NNT results are indicative of a more effective treatment. The NNT was calculated using the following formula:

$$\text{NNT}=\frac{1}{{\text{P}}_{\text{HAPWOC}}-{\text{P}}_{\text{saline}}}$$

Where P_HAPWOC_ & P_saline_ were the probability of a wound-related complication for HAPWOC and saline cohorts, respectively. The cost per NNT was calculated by multiplying the incremental cost of HAPWOC by the NNT. The benefits of the NNT and cost per NNT are its: ability to be easily understood, straightforward calculation, and it is less sensitive to event rates in the control group (saline).

Modeling strategies followed recommended practices by the International Society of Pharmacoeconomics and Outcomes Research and the Society of Medical Decision Making.^9^ Due to the short-time horizon, the lack of time dependent repeated events and health states, a state transition model was not approprirate.^10^ The model was developed using Treeage Software (Williamstown, MA). The base case results were verified through the development of an identical model in Excel (Microsoft, Redmond, WA). All data used in the model was publicly available in the literature.

### Parameter Estimates

The clinical and utilization data were obtained from a single-center prospective study previously published (Hiebert, 2016). All patients followed the same treatment protocol with the exception of the HAPWOC cohort, who received HAPWOC irrigation rather than saline. Wound complications were assessed at 1-, 7-, and 14-days post debridement. The presence of complication at any time was used to inform the overall complication rate for both cohorts. The incremental cost of HAPWOC was the only difference in utilization between cohorts. The cost of HAPWOC was the total cost of materials per the application of irrigation during the debridement procedure and provided by the manufacturer. Table 1 lists the key model inputs and their values used in the model.

**Table 1.** Model Parameters

	**Base**	**Low**	**High**	**Distribution**	**Reference**
HAPWOC Cost/Unit	$50	$37.50	$62.50	Gamma	^11^
HAPWOC Units per Procedure	1	1	3	Uniform	^11^
					
**Complications**					
HAPWOC	25%	18.75%	31.25%	Beta	^7^
Saline	80%	60.00%	100.00%	Beta	^7^

Abbreviations: HAPWOC, hypochlorous acid preserved wound cleanser.

### Sensitivity Analysis

One-way deterministic and probabilistic sensitivity analyses were performed to gauge the reliability and robustness of the results of the ICER to changes in model input parameters. The one-way sensitivity analysis varied key model parameters one-by-one and recalculated the ICER each time. The probabilistic sensitivity analysis used a Monte-Carlo approach to recalculate the ICER over 10 000 simulated patient trials. All parameters were varied +/- 25% from the base case value to derive the sensitivity range.

## RESULTS

Table 2 reports the results of the cost-effective analysis for HAPWOC versus saline irrigation. In the base case model, the ICER for HAPWOC was US$90.85 per wound-related complication avoided. This means that compared to saline, one would be expected to spend US$90.85 to avoid one additional wound-related complication with HAPWOC. The incremental expected cost was US$49.97 and an incremental effect was a 55% decreased probability of a wound-related complication for the HAPWOC cohort. For any one patient, there would be an incremental cost of nearly US$50 and a corresponding decreased probability of avoiding a wound-related complication of 55%. The primary model was verified using Microsoft Excel. The results were duplicated exactly and provided confidence that no formula errors existed.

**Table 2.** Cost-Effectiveness Analysis Model Results

	**Cost**	**Incremental Cost**	**Effect**	**Incremental Effect**	**ICER**
Saline	$0.00	-	0.20	-	-
HAPWOC	$49.97	$49.97	0.75	0.55	$90.85

Abbreviations: HAPWOC, hypochlorous acid preserved wound cleanser; ICER, incremental cost-effectiveness ratio.

The ICER represents the cost to avoid one-wound related complication using HAPWOC.

The NNT to avoid one wound-related complication was two (Table 3) indicating that for every two patients treated with HAPWOC, one wound-related complication would be avoided. The cost per NNT was US$99.94, which indicated that for less than US$100 in additional expected treatment costs over two patients, one wound-related complication will be avoided.

**Table 3.** NNT Results

	**Cost**
NNT	2
Cost per NNT	$99.94

Abbreviations: Number needed to treat, NNT.

The NNT is rounded up to the nearest whole number to reflect actual patients needed to treat to avoid one wound-related complication. The cost per NNT reflects the expected costs to avoid one wound-related complication.

**Figure 2.**
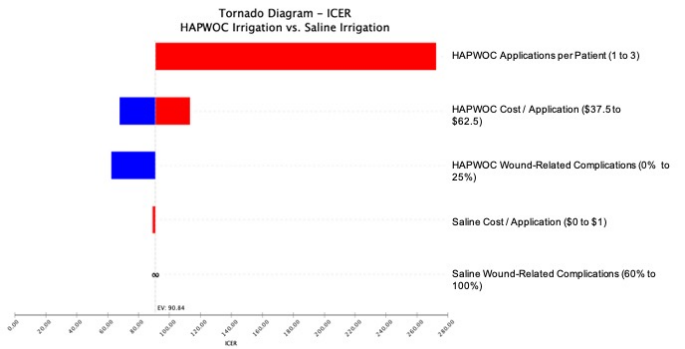
Tornado Diagram of One-Way Sensitivity Results Abbreviations: HAPWOC, hypochlorous acid preserved wound cleanser; ICER, incremental cost-effectiveness ratio. The tornado diagram is a visual representation of the one-way sensitivity analysis. The one-way sensitivity analysis assessed which model parameters were most important to the ICER results. Shown, here the additional use of HAPWOC influenced the ICER the most.

**Figure 3.**
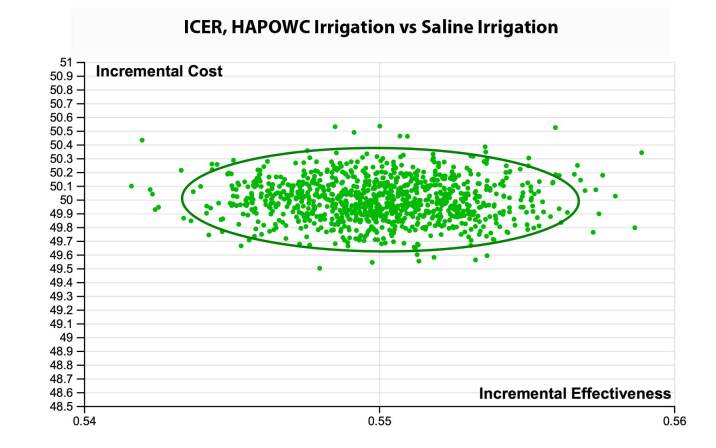
Probabilistic Sensitivity Results Abbreviations: HAPWOC, hypochlorous acid preserved wound cleanser; ICER, incremental cost-effectiveness ratio. The scatter plot shows the results of 10 000 simulated patients. Parameter values were randomly assigned based upon the data contained in Table 1 to calculate the ICER of using HAPWOC.

**Figure 4.**
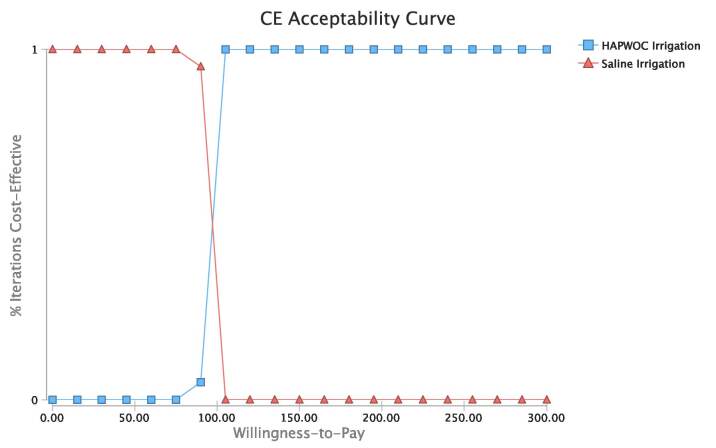
Cost-Effectiveness Acceptability Curve Abbreviations: CE, cost effectiveness; HAPWOC, hypochlorous acid preserved wound cleanser; WTP, willingness-to-pay. The acceptability curve shows the point at which HAPWOC becomes the preferred strategy ($90.85) based on the WTP amount to avoid one wound-related complication. Assuming a wound-related complication costs $91 or more, HAPWOC would be the cost-effective irrigation adjunct for low-frequency ultrasound debridement.

One-way sensitivity analysis revealed the ICER to be most sensitive to the number of units of HAPWOC used during the debridement and the cost of HAPWOC (Figure 2). The probabilistic sensitivity analysis revealed that all 10 000 simulated patient trials fell below a willingness-to-pay (WTP) threshold of US$100 per wound-related complication avoided (Figure 3). Specifically, Figure 4 illustrates the cost at which HAPWOC becomes cost-effective (US$90.85) relative to saline. This leads to the conclusion that assuming one is willing to pay US$91 or more to avoid a single wound-related complication for a patient who matched the patients reported in this study, HAPWOC was the preferred irrigation modality.

## DISCUSSION

Assessing cost-effectiveness when effects are measured in natural units (complications avoided) requires an assessment of the WTP to avoid one complication. Stated another way, was the expected cost of using HAPWOC less than the expected cost of a wound-related complication? A recent study of wound complications estimated a cost range between US$366 for a minor venous wound complication to US$7308 for a diabetic foot ulcer complication.^12^ This is a conservative estimate as a full failure and necrosis of a pedicle flap is bound to cost much more. Therefore, the ICER of US$90.85 found in this study would be considered highly cost-effective and was robust to sensitivity analysis at any cost of a complication below US$300.

Using clinical data from Hiebert and Robson (2016), we extrapolated the total expected savings of using HAPWOC.^7^ The total expected costs of using HAPWOC for all 17 patients was US$850. The total expected costs of wound-related complications conservatively ranged from US$1830 to US$36 540 (5 observed wound-related complications multiplied by US$366 and US$7308).^12^ Thus, the total savings to the health-care system would range from US$980 to US$35 690 if HAPWOC was used in conjunction with low-frequency ultrasound debridement for all 17 patients.

The use of cost-effectiveness analyses provides a common framework for comparing medical interventions for wound care. Health-care decision makers have scarce resources and must maximize the outcomes gained relative to the costs incurred. This study adds to the growing body of literature indicating HAPWOC is a cost-effective and clinically beneficial adjunct for the treatment of complex wounds.^13–15^

In the context of other wound care modalities, the use of HAPWOC appeared to provide a better value for patients with serious and complex wounds. HAPWOC was found to have a favorable ICER compared to the use of enhanced nursing best practices to avoid wound-related issues (HAPWOC ICER US$91 vs. Enhanced Nursing ICER of US$2142).^16^ Becaplermin gel was found to have an ICER of US$298 per pressure injury avoided.^17^ Whereas, the use of digital subtraction angiography for all diabetic-foot ulcers had an ICER of US$75 824.^18^

### Limitations

This study has several limitations that must be noted. First, the results are based on a small study of complex wound patients seeking treatment at a single center. As such the results may not be generalizable to other wound care centers following different treatment protocols. For instance, no adverse events occurred and, therefore, no additional costs were incurred beyond the use of HAPWOC. We provided the NNT and cost per NNT to address the small sample size and show the relatively few people necessary to treat to achieve a cost-effective outcome. Second, the clinical study effect measure was a broad definition of wound-related complication, ranging from minor to major. A conservative approach to assessing the cost-effectiveness was employed to mitigate this limitation. If the composition of complications were more likely to be major, the results of this study would be stronger for HAPWOC. Third, the clinical study used in this analysis only followed patients for 14 days with respect to any wound-related complication. Finally, this study did not include the cost of therapy, ancillary wound-related services, or lost productivity. If these costs were to be included, the results would be more favorable for HAPWOC due to fewer wound-related complications. Despite these limitations, these results provide insight into the type of irrigation used during a mechanical debridement procedure for complex wounds. A relatively minor cost of adding HAPWOC was shown to provide substantial value with respect to avoiding the high probability of an expensive wound-related complication when using saline for irrigation.

## CONCLUSION

HAPWOC was expected to be a cost-effective strategy for the treatment of complex wounds during low-frequency ultrasonic mechanical debridement. Assuming a WTP of US$100 or more to avoid a wound-related complication, HAPWOC was the preferred irrigation modality in the treatment of complex wounds that were closed definitively using WBP techniques to allow the surgical closure to be adopted. Estimated health-care system savings among the 17 patients ranged from US$980 to US$35 690. The adoption of HAPWOC should be considered a value-added adjunct to low-frequency ultrasound debridement of complex wounds.
